# Editorial: Using artificial intelligence technology for language learning

**DOI:** 10.3389/fpsyg.2023.1287667

**Published:** 2023-09-20

**Authors:** Bin Zou, Hayo Reinders, Michael Thomas, David Barr

**Affiliations:** ^1^Department of Applied Linguistics, School of Humanities and Social Sciences, Xi'an Jiaotong-Liverpool University, Suzhou, China; ^2^School of Liberal Arts, Anaheim University, Anaheim, CA, United States; ^3^Centre for Educational Research (CERES), School of Education, Liverpool John Moores University, Liverpool, United Kingdom; ^4^School of Education, Ulster University, Coleraine, United Kingdom

**Keywords:** artificial intelligence, language learning, assessment, speaking, reading

Although research on Artificial Intelligence (AI) stretches back several decades, it has become increasingly prominent over the last several years due to the emergence of Large Language Models (LLMs) and chatbots such as ChatGPT that aim to mimic human conversation and dialogue. This has led to renewed interest in the potential of AI to aid education, particularly through its potential to improve personalized learning, as well as the challenges it presents to academic integrity, and general concerns over security and privacy. In second language acquisition, recent developments in AI build on previous work in Intelligent Computer-Assisted Language Learning (iCALL) (Schulze, 2008), and aims to investigate how learning can be enhanced through personalized learning materials, instruction and feedback (Hellmich and Vinall, 2021; Xiao and Park, 2021; Dai and Wu, 2023). In the area of feedback, for example, language learners can use AI technology to record their voice on their computers or mobile devices and then receive a score and feedback through a speech evaluation system (Dizon, 2020; Zou et al., 2023a). In addition, learning outcomes can be enhanced through collaborative activities in social networking contexts when they use AI for speaking practice (Zou et al., 2023b). Beyond direct instruction, AI has made significant strides in making administrative, curriculum development and testing processes more efficient (Xu et al., 2021).

Although AI has considerable potential in language education, there is a dearth of research on the subject and the research that does exist has started to identify important areas for further investigation. One emerging concern is how to provide students with a range of course- and assessment-aligned input. Research has identified gaps in the programming of AI for language learning, consisting of adapting features such as various types of instant feedback of language skills to sustain independent study. Therefore, the potential of AI programs needs be investigated further to explore how AI can be applied to foster language skills that are required in real-life contexts. It is also important to consider how instant feedback in the AI programs can meet learners' language learning goals and the ways to design a variety of feedback to enhance learners' independent study on their computers or mobile devices.

This Research Topic issue examines the opportunities and challenges of using AI to develop language skills in language learning contexts. It explores how AI can engage learners' development of language skills and the potential benefits of AI for engaging students' language practice. The five papers published in this Research Topic were subject to a rigorous process involving multiple revisions prior to acceptance for publication. The first research paper (Huang et al.) looks at using AI for reading practice with deep-learning technologies. The authors provide effective examples for teachers and designers to consider when integrating or designing such AI systems to help learners practice reading skills. The second article (Liu et al.) investigates students' perceptions of using AI apps for EFL speaking practice. Through an experimental study, the authors show the effectiveness of using AI for overall speaking skills. The third research paper (Yang et al.) focuses on using an automated writing evaluation system via Natural Language Understanding tasks to develop students' EFL writing skills in argumentative essays. Their findings demonstrate that their combined model is effective for improving learners' writing skills. The following article (Qin et al.) explores an automatic error type annotation platform for dictation practice for French as a foreign language (FFL) learning. Their study displays a positive result in using this AI system for FFL learning. The final paper (Sun) uses an experimental approach to investigate the effectiveness of utilizing peer correction when using AI for EFL speaking practice. The findings reveal that peer correction can reinforce students' learning outcomes during their practice on AI speaking tools. Thus, the author suggests considering peer correction tasks during the use of AI for EFL speaking practice.

Overall, the articles that are part of this Research Topic demonstrate that AI offers tremendous real-world potential for enhancing many aspects of language learning and teaching. We hope together the articles will inspire others to continue to investigate this important and rapidly developing field.

## Author contributions

BZ: Conceptualization, Project administration, Writing—original draft. HR: Conceptualization, Writing—review and editing. MT: Conceptualization, Writing—review and editing. DB: Conceptualization, Writing—review and editing.
